# A pilot study for a non-invasive system for detection of malignancy in canine subcutaneous and cutaneous masses using machine learning

**DOI:** 10.3389/fvets.2023.1109188

**Published:** 2023-01-26

**Authors:** Gillian Dank, Tali Buber, Gabriel Polliack, Gal Aviram, Anna Rice, Amir Yehudayoff, Michael S. Kent

**Affiliations:** ^1^Koret School of Veterinary Medicine, Hebrew University, Rehovot, Israel; ^2^HT BioImaging LTD, Hod Hasharon, Israel; ^3^Department Biomedical Engineering, Tel Aviv University, Tel Aviv, Israel; ^4^Department of Mathematics, Technion, Haifa, Israel; ^5^Department of Surgical and Radiological Sciences, School of Veterinary Medicine, University of California, Davis, Davis, CA, United States

**Keywords:** dogs, oncology, machine learning, diagnosis, artificial intelligence, neoplasia, screening test

## Abstract

**Introduction:**

Early diagnosis of cancer enhances treatment planning and improves prognosis. Many masses presenting to veterinary clinics are difficult to diagnose without using invasive, time-consuming, and costly tests. Our objective was to perform a preliminary proof-of-concept for the HT Vista device, a novel artificial intelligence-based thermal imaging system, developed and designed to differentiate benign from malignant, cutaneous and subcutaneous masses in dogs.

**Methods:**

Forty-five dogs with a total of 69 masses were recruited. Each mass was clipped and heated by the HT Vista device. The heat emitted by the mass and its adjacent healthy tissue was automatically recorded using a built-in thermal camera. The thermal data from both areas were subsequently analyzed using an Artificial Intelligence algorithm. Cytology and/or biopsy results were later compared to the results obtained from the HT Vista system and used to train the algorithm. Validation was done using a “Leave One Out” cross-validation to determine the algorithm's performance.

**Results:**

The accuracy, sensitivity, specificity, positive predictive value, and negative predictive value of the system were 90%, 93%, 88%, 83%, and 95%, respectively for all masses.

**Conclusion:**

We propose that this novel system, with further development, could be used to provide a decision-support tool enabling clinicians to differentiate between benign lesions and those requiring additional diagnostics. Our study also provides a proof-of-concept for ongoing prospective trials for cancer diagnosis using advanced thermodynamics and machine learning procedures in companion dogs.

## Introduction

Cancer is the leading cause of death in 45–47% of dogs over 10 years of age (1, 2). Cancer diagnosis is of key importance in treatment planning and providing better treatment. The ability to easily diagnose early-stage neoplasia in general practices should improve prognosis dramatically. Currently, either fine-needle aspiration or biopsies are the recommended diagnostic tests for subcutaneous and cutaneous masses. In many cases, these procedures are easily performed; however, they may be highly invasive and costly, which can delay an owner's decision to pursue a diagnostic workup. Studies that have compared the diagnostic accuracy for the diagnosis of neoplasia of fine-needle aspirates to histopathologic results showed a negative predictive value (NPV) of 63.63 and 68.7% in reviewed series in dogs and cats (3, 4). These results demonstrate the need for an alternative non-invasive procedure for early cancer detection. The HT Vista device is based on the differences between malignant and normal tissue properties, primarily the fact that both tissues display different heat transfer rates (5, 6). These thermophysical properties are affected by the differences between the compositions, morphology, and vascular networks of the tissues (7–10). The calculated rate at which heat transfers throughout a material is termed thermal diffusivity. This diffusivity is determined by three major properties: thermal conductivity, heat capacity, and density (11). In the case of living tissues, their metabolism and blood flow dramatically affect their heat transfer (12, 13). In well-established tumor tissue, characterized by increased metabolic activity, faster growth processes, and increased blood vessel generation and usage, an increase of roughly one degree Celsius, compared to healthy neighboring tissue, was reported (14). This further supports the premise that cancer cells have different thermal properties compared to normal tissues.

In this study, we hypothesize that thermal diffusivity will differ between malignant and benign canine subcutaneous and cutaneous masses and that the HT Vista algorithm would be able to differentiate these masses into either the benign or malignant categories.

## Materials and methods

### Study design and case collection

This is a prospective study that was approved by the ethical review board committee (HU-NER-2020-015-A). The study population included dogs that presented to the Veterinary Teaching Hospital at the Koret School of Veterinary Medicine (Rishon LeZion, Israel). Informed consent was obtained from the owners of all the dogs prior to enrollment in the study.

The inclusion criteria included a signed owner consent, an externally accessible subcutaneous or cutaneous mass or lymph node that could be palpated, measured, and imaged by the device and considered safe for the dog to undergo an aspirate or biopsy. Dogs were excluded from the study if they had no gross disease, the mass was inflamed or infected, or if the mass was larger than 15 cm. In addition, the case was excluded if the cytology or biopsy did not provide a diagnosis, or if the thermal imaging was not successful. All dogs were monitored for adverse effects.

The data acquired by the HT Vista system did not influence any subsequent treatment or decision-making, and the clinician and the pathology lab were blinded to the results obtained by the system. Demographic information, as well as tumor measurements and location, were recorded using standard manual case reporting forms.

### The device

The HT Vista system (hereafter termed “the system”) is based on a continuous measurement of heat diffusion through the tissue. The system is composed of a control unit which includes a mini personal computer with internet capabilities, a touch screen, a dedicated software application, and a handheld probe. The probe consists of an optical camera, a high-power LED (Light-Emitting Diode) emitter (i.e., the heating source), and an inherent LWIR (long-wave-infra-red) thermal video camera, which records the temperature throughout the scan.

### Patient preparation and testing process

The dogs were manually restrained, and the mass area was clipped. The probe was positioned above the examined area, which was subsequently scanned. The scan lasted 60 s. This included both heating of the target area by the high-power LED emitter by seven degrees Celsius for 10 s and continuous recording of the heat emitted by the tissue during and post-heating by the LWIR video camera. Then, the clinician marked two areas on an optical image of the scanned area, presented on the touch screen. The first selection represented the mass area (i.e., “site”), while the second one, adjacent to the mass, represented a normal tissue (i.e., “control”). If there were areas with different pigmentation, areas with the same pigmentation were marked. Then, unique thermal signals were produced, showing the changes in temperature in the site and the control throughout the test, based on the selection of healthy and suspicious sites by the clinician. The data obtained were uploaded to the HT Bioimaging cloud and analyzed using signal analysis techniques and a dedicated HT machine learning algorithm. The clinician was blinded to the results. Finally, the tested mass was aspirated and/or biopsied, according to the clinical recommendations. The aspirates were performed with a 25 gauge needle and submitted to an external pathology laboratory. The biopsies were performed by the surgery department and submitted to an external laboratory. Both the clinical pathologists and anatomic pathologists examining the samples were blinded to the results.

### Dataset description

Both marked sites were presented as areas of 1.5 X 1.5 mm^2^. Each was composed of a 25 pixels grid. The thermal signal of each of the pixels in both sites was represented by a set of ca. 1,000 signal descriptive features (i.e., values). These extracted features were based on mathematical, physical, and thermal properties, such as coefficients of the Pennes equation, as well as properties derived from signal analysis [e.g., Fourier series coefficients, used to describe periodic signals (15)]. The features of the control site pixels were integrated into the features of the mass site pixels, ensuring that the differences between the tissues were considered. All features were normalized to eliminate possible variances between patients and anatomical areas. Next, the control site pixels were removed from the dataset, resulting in a dataset of >1,000 normalized feature values, for each of the 25 mass site pixels, per patient. Finally, the results obtained from the cytology and/or histopathology of the mass were used to label the site as malignant or benign for subsequent training of the HT Vista algorithm.

### Training and validation procedure

The final training procedure was performed on a set of the four most important features that best differentiated between benign and malignant lesions, including two Fourier Series coefficients and a fitted decay function coefficient. Sites were labeled as malignant or benign, as described above. The data were trained using a Support Vector Machine (SVM) classifier, a widely used AI classification algorithm. The training and validation were done using a “Leave-One-Out” cross-validation procedure to demonstrate that the classifier represents a general pattern. Cross-validation is a well-established practice in machine learning for model performance evaluation on limited data. This procedure partitions the data into *N* subsets, iteratively training the classifier on *N-1* different subsets in each iteration. Then, it uses the one left-out subset as a test set (i.e., classifies the single subset it was not trained on as malignant or benign). In this procedure, all instances are eventually used as both training and test sets. Commonly, multiple iterations of cross-validation are performed, and performance assessments are averaged over all iterations to increase robustness and reduce variability (16–18). Specifically, the “Leave-One-Out” cross-validation withholds in each iteration a single set of pixels belonging to the same mass (i.e., the same patient), while the other masses and their pathology results train the classifier. The trained classifier was then applied to the data of the one mass left out, resulting in a classification of the marked tested area as either high-risk or low-risk (i.e., malignant or benign).

After classifying all lesions, the performance of the algorithm was assessed using a confusion matrix. The matrix summarizes the identities and differences between the real diagnosis obtained from cytology or histopathology and the predictions made by the algorithm. Each cell of the matrix holds the number of correct and incorrect classifications made by the algorithm of each of the possible classes. That is, the matrix counts how many true-positives (malignant lesions classified as malignant), true-negative (benign lesions classified as benign), false-positive (benign lesions classified as malignant), and false-negative (malignant lesions classified as benign) cases were found in the study. The overall performance of the HT algorithm was then assessed by calculating five measures: (7) Accuracy–the overall fraction of correct classifications. (1) Sensitivity- the fraction of high-risk predicted lesions within the malignant or premalignant pathology reports. (3) Specificity–the fraction of low-risk predicted lesions within the benign or non-malignant pathology reports. (4) Positive predictive value (PPV)–the fraction of true positives within the high-risk predictions. (5) Negative predictive value (NPV)–the fraction of true negatives within the low-risk predictions.

## Results

Forty-nine dogs were initially included in the study. A total of four dogs were excluded: one mass was not diagnosed, one was not sufficiently clipped for the scan to be diagnostic, and two did not have healthy areas imaged during the scan. A final group of 45 dogs met the inclusion criteria. Thirty-three were mixed-breed dogs, and 12 were purebred dogs. No purebred dog was over-represented. There were 16 intact female dogs, three spayed female dogs, and 26 intact male dogs. The median age was 11 years, ranging between four and 14.

Of the 45 dogs, 24 had one lesion sampled, 18 dogs had two lesions sampled, and three dogs had three lesions sampled, resulting in a total of 69 lesions. Twenty dogs were classified with 27 malignant lesions based on their cytology or histopathological diagnosis. Forty-eight lesions were diagnosed using cytology, and 21 lesions were diagnosed using histopathology (Table 1).

**Table 1:** Classification of cytology and histopathology results.

**Tumor type**				
**Benign**		Cytology	Histopathology	Total
	Adenoma	1	3	4
	Benign epithelial/adnexal cyst/tumor	4	1	5
	Benign melanoma		1	1
	Lipoma	20	1	21
	Papilloma	1		1
	Perineal adenoma	1	1	2
	Plasmacytoma		1	1
	Reactive lymph node	2		2
	Sebaceous adenoma	3		3
	Sebaceous hamartoma		1	1
	Sebaceous hyperplasia	1		1
**Benign Total**		33	9	42
**Malignant**		Cytology	Histopathology	Total
	Adenocarcinoma		3	3
	Cutaneous hemangiosarcoma		3	3
	Lymphoma	10		10
	Mast cell tumor	3	2	5
	Metastatic lymph node (hemangiosarcoma)	1		1
	Metastatic lymph node (melanoma)		1	1
	Osteosarcoma		1	1
	Soft tissue sarcoma		1	1
	Undifferentiated neoplasia		2	2
**Malignant total**		14	13	27

Using the machine learning classifier, each examined site was classified as either high-risk or low-risk for malignancy, and results were compared to the pathology reports. These results are shown in Table 2. In total, 62 out of 69 lesions were correctly classified, 25 as malignant and 37 as benign, while seven were misclassified. Five were false-positive classifications, including one keratinous cyst, three deep lipomas, and one sebaceous gland adenoma. The other two were false-negative cases which included two lymphomas. The overall accuracy, sensitivity, specificity, positive predictive value, and negative predictive value were 90, 93, 88, 83, and 95%, respectively.

**Table 2:** Results of the HT Vista system classification for all sites.

		**HT classification**		
**Benign/malignant**	**Tumor type**	**Benign**	**Malignant**	**Total**
	Adenoma	4		4
	Benign epithelial/adnexal cyst/tumor	4	1	5
	Benign melanoma	1		1
	Lipoma	18	3	21
	Papilloma	1		1
	Perineal adenoma	2		2
	Plasmacytoma	1		1
	Reactive lymph node	2		2
	Sebaceous adenoma	2	1	3
	Sebaceous hamartoma	1		1
	Sebaceous hyperplasia	1		1
**Benign total**		37	5	42
				
		**Benign**	**Malignant**	**Total**
	Adenocarcinoma		3	3
	Cutaneous hemangiosarcoma		3	3
	Lymphoma	2	8	10
	Mast cell tumor		5	5
	Metastatic lymph node (hemangiosarcoma)		1	1
	Metastatic lymph node (melanoma)		1	1
	Osteosarcoma		1	1
	Soft tissue sarcoma		1	1
	Undifferentiated neoplasia		2	2
**Malignant total**		2	25	27

## Discussion

AI-driven medical devices are becoming more and more common in veterinary medicine. They are used to solve problems of high logical or algorithmic complexity, ranging from diagnosis and disease detection to making reliable predictions and reducing medical errors (19). In this study, we introduced a novel diagnostic AI-based imaging system, the HT Vista, which aims to provide a high degree of accuracy in differentiating between benign and malignant, cutaneous and subcutaneous lesions in dogs, based on the response of a tissue to thermal excitation. Malignant tumor tissues differ from normal tissues by their known high metabolic rate and increased perfusion and their capability to transfer heat, which in turn shows a different response to thermal excitation (11–14). Additional factors that have been reported to influence thermal imaging in dogs include inflammation, infection, trauma and temperature at the time of the scan (20). However, our results do not appear to have been influenced by these factors. We found that the temperatures recorded during the thermal relaxation phase distinguished between normal and malignant tissues. An AI-based algorithm was trained on physical and thermal features, using samples labeled as malignant or benign based on the blinded pathological results.

Our study included a wide range of both benign and malignant tumors. The overall accuracy of the system was 90%, correctly classifying 62 out of 69 masses, with only two false negatives (lymph nodes diagnosed with lymphoma). The explanation for the misclassification of these cases was most likely the different anatomic structures of lymph nodes, which might have resulted in inadequate heating to elicit a representative thermal signal.

Five were false-positive classifications, including one keratinous cyst, three deep lipomas, and one sebaceous gland adenoma. Several explanations for these false results include that deep inhabiting tumors may require a change of the heat source configuration (e.g., the wavelength and penetration characteristics) and that the liquid content within cysts may heat differently. In any case, positive results should cause the clinician to continue to diagnose the mass, which will lead the clinician to conclude that this is a benign mass in cases of false positives. This is preferable to a higher number of false negatives, which would cause clinicians to send home animals with malignant tumors.

The HT Vista system's algorithm was programmed to give a high degree of certainty in classifying a mass as benign, thus minimizing the risk of false-negative cases. Therefore, in high-risk cases, this system enables the clinician to recommend continuing to work up these masses with either an aspiration and /or a biopsy and not take the wait-and-see approach.

Factors that influence cancer detection include inflammation and infection, which can cause dysplasia and lead to false positive results on the fine needle aspirates. In this study, there was one case of sebaceous hyperplasia that was a true negative based on the classifier. Additional causes that may influence detection include acellular samples, as can occur in cases of lipomas and sarcomas. In this study, the device accurately classified 18/21 lipomas and all carcinomas and sarcomas.

In this study, the performance of the algorithm's classifier was assessed using a “Leave-One-Out” cross-validation method, which is cross-validation taken to its extreme. This method is useful for evaluating machine learning models with a limited data set, as in our study, and provides an accurate and unbiased estimate of model performance (21).

The limitations of the study include the low number of cases and that the deeper tumors, including both deep lipomas and lymph nodes, may require changing the heat source configuration, as previously mentioned. In addition, cutaneous and epidermal tumors do not always present the same way or have the same disease progression as mesenchymal tumor types, which may cause a variation in the thermal signal. Skin pigmentation was not shown to have an effect on the thermal heating in this study, however, should be further assessed in future studies with additional dogs with different pigmentation. Additionally, larger studies should help give an understanding whether these differences affect thermal diffusivity.

Future directions include an additional multi-center trial with a larger study population to validate the system. As machine learning accuracy improves with additional data, the HT Vista's algorithm is expected to improve its analytic capabilities. Therefore, it is expected to provide more accurate results in the future.

In conclusion, in this study, we showed a proof of concept of a novel non-invasive diagnostic method and decision support tool for the clinical management of cutaneous and subcutaneous masses in dogs, using dynamic heat diffusivity and analysis of the produced signal utilizing advanced machine learning.

## Data availability statement

The raw data supporting the conclusions of this article will be made available by the authors, without undue reservation.

## Ethics statement

The animal study was reviewed and approved by Hebrew University HU-NER-2020-015-A. Written informed consent was obtained from the owners for the participation of their animals in this study.

## Author contributions

All authors contributed to the study-including the study planning, case accrual, algorithm, and writing the manuscript. All authors contributed to the article and approved the submitted version.
